# Modeling neoplastic disease with spheroids and organoids

**DOI:** 10.1186/s13045-020-00931-0

**Published:** 2020-07-16

**Authors:** Michele Zanoni, Michela Cortesi, Alice Zamagni, Chiara Arienti, Sara Pignatta, Anna Tesei

**Affiliations:** Biosciences Laboratory, Istituto Scientifico Romagnolo per lo Studio e la Cura dei Tumori (IRST) IRCCS, Meldola, Italy

**Keywords:** Cancer, 3D models, Tumor microenvironment, Organoid, Spheroid, Drug discovery

## Abstract

Cancer is a complex disease in which both genetic defects and microenvironmental components contribute to the development, progression, and metastasization of disease, representing major hurdles in the identification of more effective and safer treatment regimens for patients. Three-dimensional (3D) models are changing the paradigm of preclinical cancer research as they more closely resemble the complex tissue environment and architecture found in clinical tumors than in bidimensional (2D) cell cultures. Among 3D models, spheroids and organoids represent the most versatile and promising models in that they are capable of recapitulating the heterogeneity and pathophysiology of human cancers and of filling the gap between conventional 2D in vitro testing and animal models. Such 3D systems represent a powerful tool for studying cancer biology, enabling us to model the dynamic evolution of neoplastic disease from the early stages to metastatic dissemination and the interactions with the microenvironment. Spheroids and organoids have recently been used in the field of drug discovery and personalized medicine. The combined use of 3D models could potentially improve the robustness and reliability of preclinical research data, reducing the need for animal testing and favoring their transition to clinical practice. In this review, we summarize the recent advances in the use of these 3D systems for cancer modeling, focusing on their innovative translational applications, looking at future challenges, and comparing them with most widely used animal models.

## Introduction

Despite the substantial progress made in the last few decades in improving cancer diagnosis, prevention, and treatment, there are still numerous obstacles to overcome to further enhance the quality of life and survival of cancer patients [1–4]. Currently, < 10% of new anticancer drug candidates entering phase I trials are eventually approved by the Food and Drug Administration (FDA) [5], with cancer having the lowest approval rate of new drugs with respect to other therapeutic areas [6]. One of the major challenges facing the development of new anticancer treatments is the transition of preclinical breakthroughs from “bench to bedside” [7].

It is generally accepted that experimental models are essential tools in the field of cancer research. However, the majority of cancer models used in drug discovery experiments poorly recapitulate the complexity and heterogeneity of human tumors [8], where both genetic and microenvironmental factors contribute to disease progression, response to therapy and metastatic spread [9].

Traditional bidimensional (2D) cell cultures initially developed by Harrison in the early 19th century [10] played an important role in establishing basic tumor biology research and continue to be routinely used thanks to their wide availability, easy handling, reproducibility, and low cost (Table 1) [11–13]. However, a major drawback of cell models are their failure to mimic the heterogeneity of original tumors [13]. Indeed, the procedures used for the generation of 2D cell cultures from primary tumor specimens are extremely inefficient, permitting the in vitro selection and expansion only of clones capable of growing in flasks or petri dishes [7, 14]. Moreover, cells cultured in vitro for several passages leads to substantial and unpredictable genetic changes [13], with a cell morphology barely resembling that observed in vivo [1, 15]. Consequently, the use of animals for experimentation has intensified, increasing the overall length and cost of the drug discovery process. Several animal models have been generated to study human cancers as they do not have some of the drawbacks of 2D cell cultures (i.e., lack of stroma, vasculature, and immune components), and among these, mice models are the most widely used.

**Table 1:** Main features of preclinical cancer models

Features	Cell culture	Spheroids	Organoids	PDXs
**Cost**	Low	Low	Medium	High
**Time**	Low^*^	Low^*^	Medium^*^	High^*^
**Easy handling**	**+++**	**++**	**+**	**-**
**Success rate**	High	High	Medium	Low
**Throughput potential**	High	High	Medium	Low
**Tumor heterogeneity**	No retention	Partial retention	Retention	Retention
**Genetic manipulation**	**+++**	**+++**	**+++**	**-**
**Human immune components**	**-**	**+**	**+**	**-**
**Tumor–stroma interaction**	**-**	**++**	**++**	**+++**

Features are scored as follows: ^*^Low (< 1 month), ^*^Medium (1–3 months), ^*^High (several months). (+++) optimal, (++) good, (+) sufficient, (-) not suitable

Transgenic mice can be generated to study genes involved in cancer development and/or to test innovative targeted therapy strategies. Moreover, patient-derived xenografts (PDXs) and tumor xenografts have been developed to more closely resemble the complexity of clinical tumors [16]. Despite the undeniable importance of animal models, it must also be underlined that they, like cell cultures, have some drawbacks. In particular, their genomic and immune profile does not exactly match that of humans [17], especially in mice PDXs where tumors may undergo mouse-specific evolution [18]. Furthermore, they are very resource- and time-consuming models to develop, and their uses are limited because of feasibility and ethical issues (Table 1).

In the past few decades, enormous progress has been made in the development of new cancer-mimicking technologies. Models such as cell co-cultures, three-dimensional (3D) cultures and patient-derived tumor organoids closely resemble tumor cytoarchitecture and also have the advantage of mimicking tumor behavior, which is heavily dependent on environmental signals, cell–cell interactions and the extracellular matrix (ECM) [19–21].

In the present review, we describe the current state-of-the-art in the use of 3D cell culture technology for cancer modeling, focusing on the potential innovative translational applications of scaffold-free 3D models and highlighting their strengths and weaknesses.

## 3D cancer modeling approaches

An ideal 3D model to study cancer biology should resemble tumor tissue-specific architecture, and its pathophysiological microenvironment where tumor cells show many of the in vivo characteristics such as proliferation, differentiation, motility, and metabolism [22]. Solid tumors develop through the interaction between multiple cell and non-cell components, exploiting mechanisms similar to those found in the early stages of developing organs [23, 24]. Accordingly, tumors are considered as abnormal organs [23] displaying several biochemical gradients (i.e., oxygen, metabolites) and different physical properties that substantially impact cell behavior, resulting in a heterogeneous response to treatment [1, 25, 26]. Tumor cells in vivo are surrounded by different cell types (i.e., immune cells, stromal cells, endothelial cells) and other extracellular components (i.e., ECM, metabolites, extracellular vesicles (EVs), growth factors, cytokines) that constitute the tumor microenvironment (TME) [23]. Taking this into account, several methods to “assemble” in vitro 3D culture models have been developed to recapitulate in vivo suboptimal growth conditions and to study in depth the multifaceted features of the dynamic microenvironment of the tumor. These methods are usually categorized into two main classes: scaffold-based and scaffold-free systems.

Although the description of scaffold-based 3D models is beyond the scope of the present review, it must be underlined that they have undeniable advantages (i.e., they can be constituted by synthetic or naturally derived biopolymers mimicking the interaction between cancer cells and the ECM, providing a structural/physical support for the 3D culture) [1]. The biochemical and mechanical properties of the materials used in these models strongly affect cellular behavior [22, 27]. Moreover, physical properties such as stiffness, porosity, and surface chemistry, but also biological properties (i.e., biodegradability and cell compatibility) vary dramatically among the wide variety of biomimetic materials available [28], often making it difficult to obtain a controlled matrix and good reproducibility of biomaterials [28]. Indeed, the main issue in using these models is the appropriate selection of the 3D scaffold materials on the basis of the desire application.

**Fig. 1:**
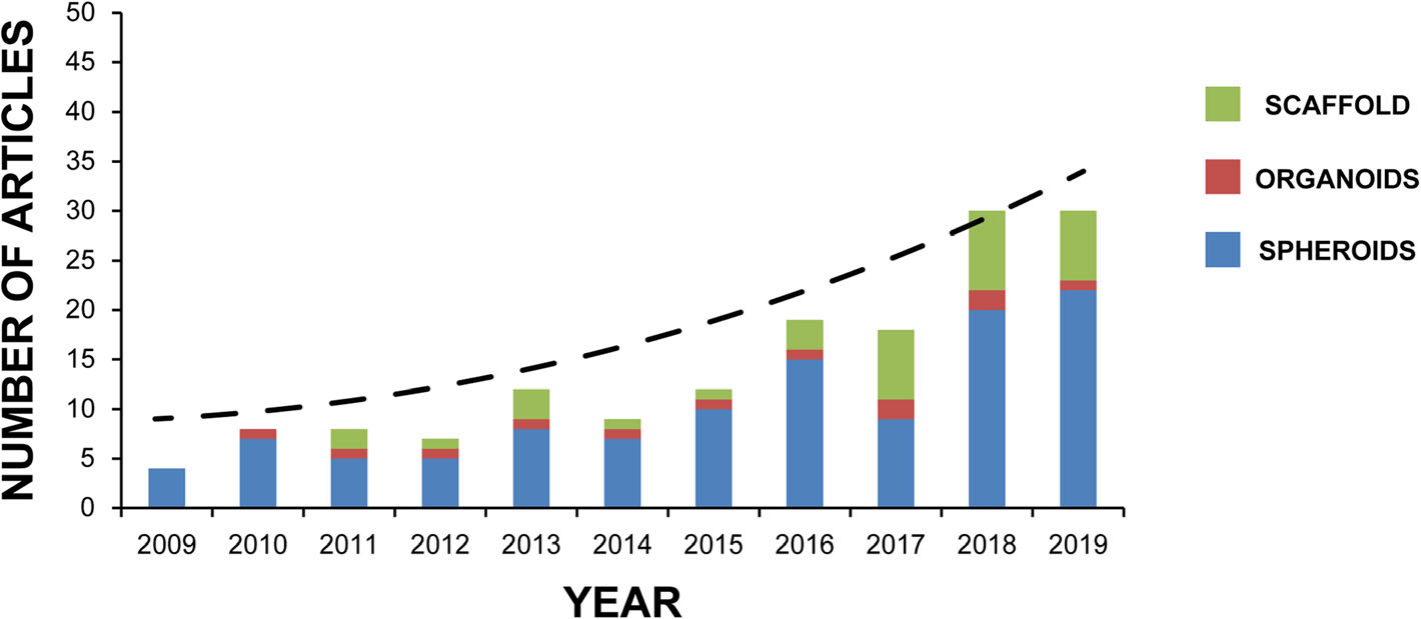
Sarcoma 3D models. Search for articles appearing in PUBMED over the past 10 years (2009–2019) using the mesh terms “tissue scaffolds” AND “sarcoma” (green); “organoids” AND “sarcoma” (red); “spheroids, cellular” OR “spheroid” AND “sarcoma” (blue)

Conversely, scaffold-free systems usually refer to self-assembled models in which cells aggregate and interact [1, 22, 29, 30]. These 3D models have emerged as a pioneering approach in the field and are being used increasingly in cancer models and drug development studies. In fact, 3D scaffold-free modeling has been widely used in solid tumors derived from several epithelial tissues, such as breast, lung, prostate, or colorectal cancer [1]. With regard to non-epithelial tumors, in particular, sarcomas, obtaining 3D models (such as spheroids or organoids) have proven to be more complicated and challenging because of the extreme heterogeneity of the cells composing them, as highlighted by Drost et al. [7]. However, great efforts made by researchers to overcome the difficulties in generating 3D sarcoma models have led to the publication of several studies using sarcoma-derived spheroids (Fig. 1, Table 2). This bodes well for the attainment of more sophisticated 3D models such as sarcoma organoids, which would represent an important step forward in the fight against one of the most feared class of tumors.

**Table 2:** 3D in vitro sarcoma models

Tumor subtype	Scaffold-free 3D models	Scaffold-based 3D models
Osteosarcoma	[31–35]	[36–38]
Chondrosarcoma	[39–42]	[43, 44]
Ewing sarcoma	[45–48]	[49–52]
Soft tissue sarcomas	[53–56]	[57–59]

Most recent and relevant references are reported

In the following sections, we will focus on the two most common models used for cancer research (i.e., spheroids and organoids), highlighting differences and discussing related strengths and weaknesses.

## Spheroid model

**Fig. 2:**
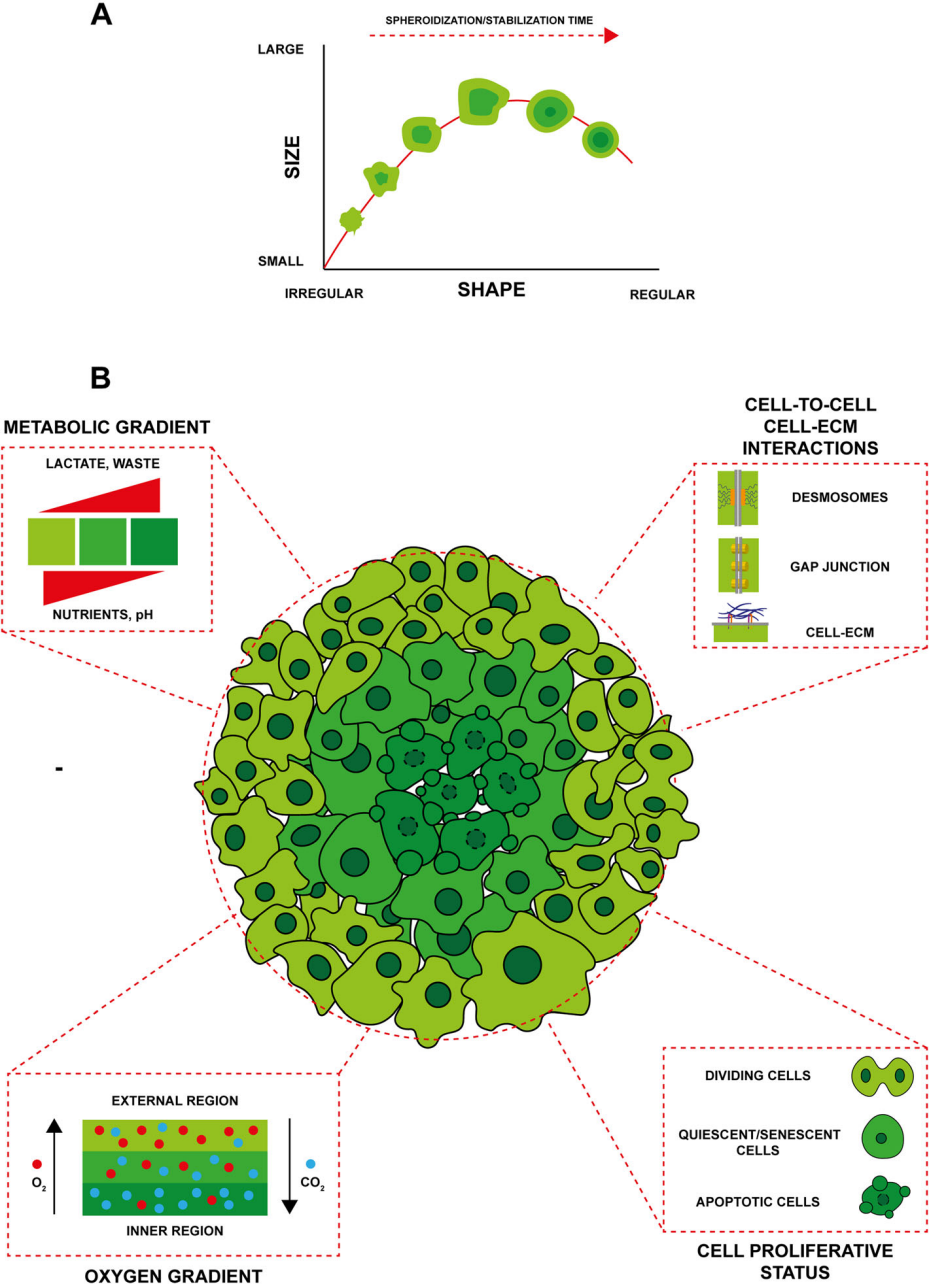
Spheroid model. a Schematic representation of spheroid variation in shape and size over time. b Main characteristics of spheroid model. The spheroid is composed of several functionally differentiated areas and layers resulting from the impaired distribution of nutrients and oxygen. Tumor cells composing the spheroids interact with each other, developing a well-organized spatial architecture characterized by differences in phenotypic, functional, and metabolic status.

Tumor spheroids, one of the most versatile and common scaffold-free models [1, 29], are typically obtained from single-cell suspensions that are self-assembling or forced to aggregate. These 3D microtissues are used to model a wide variety of tumors [60–63], reproducing, in particular, avascular tumor mass microregions [64], intervascular domains, and micrometastases [65, 66]. Tumor spheroids of appropriate dimensions (> 500 μm in diameter) resemble the complex tumor scenario as they are composed of several specialized areas and layers where cells have different phenotypic, functional, and metabolic behaviors (Fig. 2). In particular, they display a well-organized spatial architecture with an external proliferative layer, an intermediate zone composed of quiescent and senescent cells and an inner apoptotic and necrotic core resulting from the impaired distribution of nutrients and oxygen in these areas [1, 30, 67]. Spheroid volume increases exponentially in the early stages and is followed by a period of “spheroidization/stabilization” in which the spheroids reach a sort of equilibrium, becoming more regular in shape and decreasing in volume (Fig. 2) [1, 29]. This latter phase is important for the development of the functional and structural organization of the spheroid itself [30, 64, 68]. Tumor cells within the spheroid closely interact with each other and such cell–cell interactions affect cancer cell behavior in terms of proliferation, survival, and response to therapy [69]. Cell–cell cohesiveness is enforced by the formation of desmosomes and dermal junctions [70] through the activation of adhesion receptors such as E-cadherins [71], and the secretion of ECM proteins and proteoglycans (Fig. 2) [72]. The close interactions between cells coupled with the deposition of several ECM proteins (collagens, fibronectin, laminin, elastin tenascin) increase spheroid density, forming a physical barrier that prevents and limits the transport of drugs into the spheroid mass [73, 74]. In addition, the increased interstitial fluid pressure inhibits the penetration and distribution of anticancer compounds by convection [75]. During spheroid growth, gradients of oxygen, metabolites, nutrients, and pH are established, strongly influencing the therapeutic effects of various drugs (Fig. 2) [69, 74]. Cancer cells under hypoxic conditions modify their metabolism, switching from oxidative phosphorylation to anaerobic glycolysis, thus obtaining energy by the conversion of pyruvate into lactate [76]. The release of lactate contributes to the acidification of the inner areas of the spheroids [77, 78], altering several cellular processes such as migration, immunomodulation, and chemo-radiotherapy resistance [79, 80]. Low pH directly affects the efficacy of some anticancer drugs (e.g., doxorubicin, vinblastine, methotrexate, anthraquinone) reducing intracellular uptake and tissue penetration through the alteration of their net charge [81–83]. The acidic microenvironment coupled with the lack of oxygen and nutrients are responsible for the quiescent/senescent state of the cancer cells in the deepest regions of the spheroid mass, the same phenomenon that occurs in in vivo tumors. Such cells continue to produce cytokines and growth factors but display a reduced proliferative state, making them more resistant to drugs usually employed to target cells with high proliferative rate (e.g., taxanes, cisplatin, oxaliplatin) [84, 85]. Conversely, the hypoxic environment contributes to the development of resistance to drugs requiring oxygen to induce cell death (e.g., 5-fluorouracil, cisplatin, and irinotecan) and radiotherapy [86].

Currently, spheroid models can be classified on the basis of the type of cancer cells and cultured methods used and on the desired application.

### Multicellular tumor spheroids

Multicellular tumor spheroids (MTS) are mainly generated from established cancer cell lines grown in conventional cell culture media supplemented with serum (Table 2) [87]. It thus follows that they can be considered an extension of the standard bidimensional culture of cancer cell lines showing a similar limited histological resemblance to the primary cancer. Their main differences with respect to bidimensional cell cultures, apart from the capacity to grow as spherical colonies in suspension culture, are that MTS replicate the metabolic and proliferative gradients of clinical tumors and show substantial multicellular chemoresistance, thus mimicking what happens in cancer patients [88]. Additional advantages of MTS over other 3D systems are cell clonality, ease of maintenance, and simplicity of genetic manipulation, making this model an appropriate tool for high-throughput drug testing [89]. Although several methods, mainly based on anchorage-independent technologies, have been developed to generate large amounts of MTS of different sizes and shapes, not all cell lines are capable of forming spheroids [90]. Indeed, each cell line and method chosen for use requires specific optimization. In particular, cell culture time and cell density are two important parameters influencing spheroid formation.

### Multicellular tumor-derived spheroids

Multicellular tumor-derived spheroids (MTDS), also called tumorspheres [87], are usually obtained from dissociated tumor tissue and represent another spheroid model widely used in cancer research (Table 3). Like MTS, MTDS have a limited histological similarity to the primary cancer from which they are derived but differ from MTS mainly in terms of their enrichment in cancer stem cells (CSCs) or cells with stem cell-related features. CSC enrichment in MTDS cultures can be confirmed by functional assays that determine the tumorigenicity of spheroids (i.e., in vivo tumor-formation assays after transplantation into immunocompromised mice), their expression of CSC-related markers (i.e., *ALDH*, *CD133*, *CD44*), and their pluripotency (i.e., ability to differentiate into tissue-specific cell lineages). From a methodological point of view, MTDS can be obtained with the same methods used to generate MTS, the main differences being the addition of specific tissue-related growth factors and the use of serum-free culture medium. Specific growth factors are used to carefully reproduce stem cell-like states [91] for serum-free culture conditions, whereas serum is avoided because it is considered a differentiating factor (Table 3) [91]. MTDS have been widely used in in vitro studies on cancer stemness, and despite some uncertainty about the link between sphere formation and CSC phenotype, they are widely regarded as a surrogate method for CSC isolation and ex vivo expansion [92–96]. In particular, it has been hypothesized that CSCs are related to chemoresistance and metastatic spread, and some authors have pointed out that CSCs are, by their very nature, more resistant to xenobiotics and drugs than many of the malignant cells composing the tumor bulk [97, 98]. Such resistance is mainly due to the increased expression of aldehyde dehydrogenase (ALDH) enzymes, which are capable of metabolizing drugs [99], enhanced DNA damage response [100], and increased activity of ATP-binding cassette (ABC) transporters [101]. Consequently, MTDS have predominantly been used to investigate CSC chemoresistance in vitro to shed light on the intrinsic drug resistance of advanced cancers.

### Integrating microenvironment: heterotypic spheroids

It is known that solid tumors are composed not only of cancer cells, but also of stromal cells such as fibroblasts (or cancer-associated fibroblasts, CAFs), immune cells, lymphatic endothelial cells, vascular endothelial cells, pericytes, and adipocytes [102]. Although these cells are not malignant, their interactions with cancer cells in the tumor milieu foster tumor angiogenesis, proliferation, invasion, and metastasis and also mediate mechanisms of drug resistance [103]. Such interactions have been amply described in both solid and hematological tumors where the activation of several crucial pathways involved in DNA repair, proteasome activation, inflammation, ECM production, invasion, and caspase inhibition are enhanced [104]. 3D tumor spheroids can be optimized by co-culturing cancer and stromal cells, such as fibroblasts [105], endothelial cells [106], or immune cells [107] to mimic the cellular heterogeneity of solid tumors and the resistance mediated by tumor–stromal cell interactions (Table 3). Of note, the direct interaction between stromal and cancer cells coupled with the release of cytokines, extracellular vesicles, and growth factors reconstitute the intricate signaling network of in vivo tumors [108]. Generally, spheroids composed of a single cell type are called homotypic spheroids, while those constituted by multiple cell types are known as heterotypic spheroids. Different stromal:cancer cell ratios have been tested to recapitulate specific tissue composition found in vivo [109]. In particular, fibroblasts represent one of the most abundant populations of stromal cells in the TME, contributing to tumor initiation, progression, metastasis, and response to therapy [108]. For this reason, heterotypic spheroids composed of tumor cells and CAFs are widely used in drug discovery studies [109–111].

**Table 3:** Main features of the most common spheroid models used in preclinical cancer research

Spheroid models	Cells of origin	Culture medium	Culture method	References
**Multicellular tumor spheroids (MTS)**	Established cancer cell lines	Conventional medium supplemented with serum	Non-adherent conditions	[87]
**Multicellular tumor-derived spheroids (MTDS)**	Cancer cells derived from dissociated tumor tissue	Medium without serum supplemented with growth factors (e.g., FGF2, EGF)	Non-adherent conditions Pre-sorting of specific cancer cell populations	[91]
**Heterotypic spheroids**	Cancer cells mixed with stromal cells and/or immune cells	Conventional medium supplemented with serum	Non-adherent conditions Physiological ratio cancer:stromal/ immune cells to mimic clinical tumors	[105–107]

## Organoid model

**Fig. 3:**
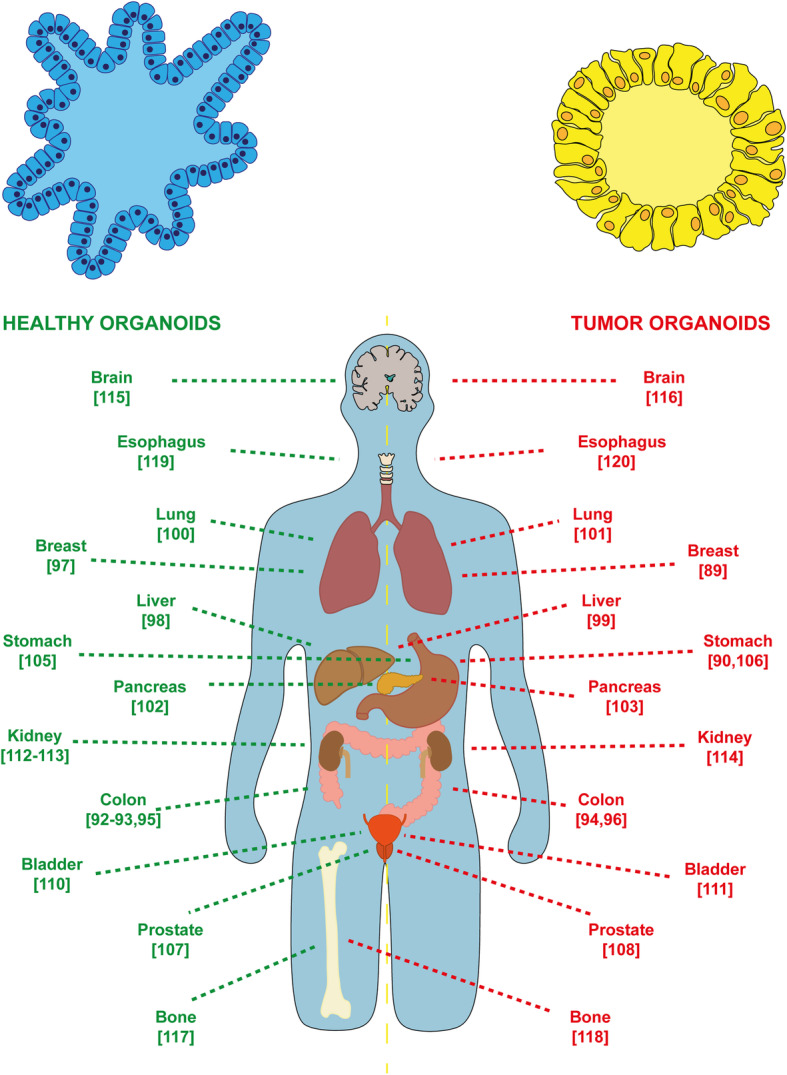
Organoid model. Organoids currently established from healthy and cancer tissues. (References are indicated in brackets)

Decades of research into developmental biology and organ physiology together with a deeper understanding of techniques for growing tissue ex vivo have improved our ability to recapitulate organogenesis cues in vitro, leading to the development of the organoid model [112, 113]. Recent years have seen a plethora of new techniques for growing tissue as 3D in vitro organotypic cultures and, in particular, for the development of disease-modelling organoids. Organoids are a powerful in vitro system and are increasingly being used in a wide range of studies. They are defined as self-organized 3D structures derived from adult or embryonic stem cells mirroring the architecture and functionality of the tissue of origin or of the tissue from which they are derived [7, 112, 114]. The organoid structure reflects specific tissue characteristics in terms of distribution of differentiated cell types, global architecture, and tissue- and cell-specific functions [114]. Long-term organotypic cultures are established by supplementing the medium with a well-defined mixture of tissue-specific growth factors without feeder layers [115]. To date, organoid cultures have been established for a wide variety of human healthy tissues as well as for patient-derived tumor specimens, obtaining the so-called “tumoroid” model [7, 116]. Tumoroids reflect the genetic and phenotypic features of tumor epithelium such as heterogeneity and 3D spatial organization [117, 118]. Numerous other features facilitate their use as a model to study cancer. In particular, being human-derived, they are not hampered by interspecies differences when used for disease modelling, which is the main drawback of using animal models. Furthermore, organoids can also be propagated in vitro and cryopreserved, facilitating the creation of an organoid biobank/library of different cancer subtypes from large numbers of patients, representing an extremely useful tool for preclinical studies [7, 116, 119, 120]. To date, long-term organoid cultures have been established from several healthy and cancer tissues (Fig. 3) including colon [121–125], breast [118, 126], liver [127, 128], lung [129, 130], pancreas [131, 132], endometrium [133], stomach [119, 134, 135], prostate [136, 137], ovary [138], bladder [139, 140], kidney [141–143], brain [144, 145], bone [146, 147], and esophagus [148, 149].

### Organoids as models for personalized medicine and drug screening

**Fig. 4:**
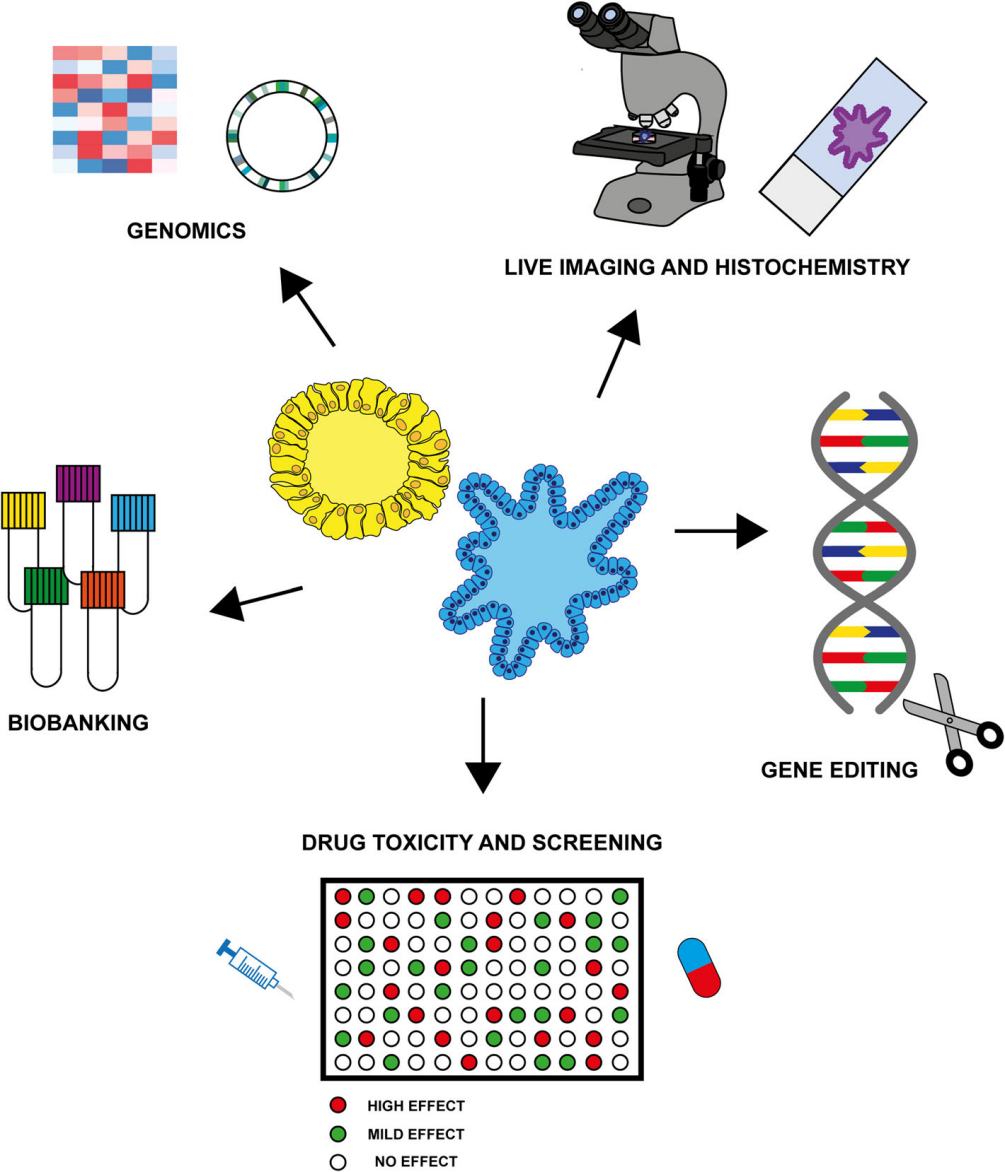
Potential research and clinical applications of organoids**.** Organoids derived from patients’ tumors with different subtypes and/or grading can be expanded and cryopreserved to create a living organoid biobank. Patient-derived organoids generated from tumors and healthy tissues can be genetically characterized and compared. They can also be used for personalized drug discovery and drug toxicity studies. Gene editing technologies can be used to study the role of mutational processes in the tumorigenesis in specific organs. Organoids resemble the heterogeneous cytoarchitecture found in vivo and advanced microscopy techniques can be used to follow the dynamic processes of organoid development and maturation

One of the main advantages of the organoid model is the possibility of obtaining healthy tissue and cancer tissue from the same patient, providing a powerful tool for predicting drug response in drug screening (Fig. 4). In particular, this model enables researchers to identify compounds preferentially targeting cancer cells rather than healthy ones, resulting in the selection of less toxic substances and thus decreasing the risk of side effects [7]. For example, liver organoids obtained from induced pluripotent stem cells (iPSCs) or healthy tissue can be used to evaluate the hepatotoxicity of new experimental drugs [150, 151] as they express fairly similar physiological levels of cytochrome P450 enzymes [152]. Similarly, iPSC-derived cardiac and kidney organoids can be used to perform toxicological studies [153, 154]. However, a major hurdle in the development of tumoroids is the overgrowth of non-tumor components present in tumor specimens, which compromises the attainment of a model homogenous for a specific genetic or phenotypic feature. For example, in colorectal cancer, mutations involved in the activation of *WNT* signaling are fairly common [123], and medium without WNT and R-spondins is needed to obtain *WNT*-mutated tumoroids [7]. Patient-derived organoids can also be used to detect epigenetic and/or genetic alterations underlying drug resistance (Fig. 4). Matching tumoroid and healthy organoid profiles can help to identify different mutation and protein patterns that could help to stratify patients for specific treatments [155]. In addition, tumor organoids derived from different regions of the same tumor have been used to study intra-tumor heterogeneity and to evaluate drug sensitivity of different tumor subclones [156].

### Organoids as a model to study tumorigenesis

Organoids can also be used to investigate the role of mutational processes in tumorigenesis. It is known that cancer results from the accumulation of several mutations in specific genes involved in different cellular processes [157]. Recently, in this context, the use of healthy organoids coupled with gene editing technologies such as CRISPR-Cas9 has led to a better understanding of organ-specific mutagenic processes [158] resulting from the accumulation of key mutations during malignant transformation (Fig. 4) [159]. For example, the introduction of a combination of driver mutations in *KRAS* (activating mutations), *APC*, *TP53*, and *SMAD4* (inactivating mutations) has been used to generate colorectal cancer (CRC) progression models [158]. When engrafted into mice, these mutated organoids grow as invasive cancer [160]. However, they spontaneously metastasize to the lungs and liver only when orthotopically transplanted into the cecum of the animals [161, 162], suggesting that advanced processes in carcinogenesis are heavily dependent on microenvironmental factors [125]. In another study, oncogenic mutations in *CDKN2A*, *KRAS*, *TP53*, and *SMAD4* introduced into human pancreatic organoids transformed normal cells into cancer cells, modelling primary and invasive pancreatic ductal adenocarcinoma when xenotransplanted in vivo [163].

Cancer organoids have also been used to model metastatic processes, in particular to investigate the different processes of invasion [164, 165]. For example, a study conducted on CRC organoids showed that inhibition of rho-associated protein kinase 2 (ROCK2) improved collective invasion in its early stages [166].

### Integrating the microenvironment in organoids: current advances

The TME plays a pivotal role in cancer progression and is crucial for tumor survival, mainly through the production and release of supporting factors [167]. In particular, the TME contributes to tumor heterogeneity, strongly affecting the adaptive cellular response which is dependent on tumor grade and stage and treatment history [168]. There are still no preclinical models that fully recapitulate patient-specific stromal, immune, structural, chemical, and molecular aspects of the heterogeneous microenvironments to which cancer cells are sequentially exposed during the course of the disease [169]. In particular, organoids usually only contain progenitors and cells of epithelial origin, lacking other cell types such as fibroblasts, immune cells, and endothelial cells [170]. After many years of disappointing results, remarkable progression has been made in cancer immunotherapy, and several organoid-based models have been created to better our understanding on how the immune system eradicates tumor cells, facilitating a personalized immunotherapeutic approach [171]. Currently, two conceptually different organoid models are used—i.e., cancer organoids cultured directly from tumors preserving endogenous immune cells and other non-epithelial cell types (holistic approach) [172–175], and cancer organoids co-cultured with immune cell subsets isolated and separately expanded (reductionist approach) [176–178]. The two are equally valid approaches to understanding cell–cell interactions, modelling immune checkpoint blockade and testing CAR-T cell-mediated cytotoxicity and represent a highly informative platform for the development of cancer immunotherapy. In a recent study, Neal et al. developed a liquid-air interface organoid system including native immune and stromal cells, enabling the reconstruction of the cytoarchitecture of different tumors [175]. The authors demonstrated that the T-cell receptor repertoire is conserved between original tumors and organoids and that the PD-1/PD-L1 immune checkpoint axis is functional in these models. In another study, murine pancreatic ductal adenocarcinoma cells were co-cultured with stromal stellate cells, the latter able to differentiate into CAFs expressing high levels of α-SMA and inflammatory mediators such as IL-6 [179]. These findings were further investigated in human organoid models where CAFs support tumor growth, creating a niche enriched in Wnt-ligands [163]. Another interesting study described the development of human blood vessel organoids using pluripotent stem cells to model diabetic vasculopathy [180]. The model was characterized by a self-assembled capillary network composed of endothelial cells and pericytes surrounded by basement membrane [116] which, in vivo, recreate a perfused system comprising arterioles and venules [180]. This model provides a new opportunity for co-culture experiments aimed at investigating the interactions between cancer cells and the vascular system. Finally, another interesting approach to fully recapitulate the physical, dynamical, cellular, and biochemical features of the TME is the integration of organoids and organ-on-a-chip technologies. The latter are defined as microfabricated cell culture devices designed to reproduce key functional features of human organs in vitro [113] including the cytostructural organization of different cell types and the dynamics of flow perfusion. Each “organ functional unit” can be interconnected through microfluidic channels simulating multiorgan interactions [181, 182] and is a good model to study the metastatic processes. Recent studies have reported the potential of combining organoids and organ-on-a-chip models, taking advantage of the best features of both systems [170, 183, 184]. An important issue for organ development and growth is the continuous supply of nutrients and oxygen usually provided in vivo by blood vessels. To mimic this, Shirure et al. developed a tumor organoid-on-a-chip system creating a 3D perfusable blood vessel network capable of delivering nutrients and/or drugs to patient-derived breast cancer organoids [185]. This platform allowed the authors to simultaneously and dynamically observe cancer cell intravasation, migration, and proliferation and also to assess the response to specific chemo- and/or targeted therapy under physiological flow conditions. The complexity of the human body is also a consequence of dynamic interconnections between its components at different levels of organization. Although organoids provide good platforms that recapitulate the features of each single organ, a system of connections between each organoid is needed to better understand systemic processes, such as metastatic spread of cancer cells or systemic cytotoxicity of anticancer drugs. Recently, Skardal et al. used a multi-organ-on-a-chip system composed of liver and heart organoids perfused in a closed loop with a micro-engineered lung tissue to evaluate the cytotoxicity of the chemotherapeutic drug bleomycin [186]. Finally, another lung-brain-liver-bone-on-a-chip model was used to study the metastatic spread of primary lung cancer to these organs, highlighting a different spatiotemporal distribution of cancer cells in each organ [187].

## Animal models: still an indispensable tool for cancer research

In vivo models to study human cancers have been widely used in cancer research for many years and represent an essential tool to better understand the complexity of cancer biology and to discover new methods of prevention, diagnosis, and treatment [188]. Many animal models can be used to recapitulate cancer more or less faithfully [189]. Less complex animal models such as *Drosophila melanogaster* [190], *Caenorhabditis elegans* [191], *Xenopus laevis* [192], and *Danio rerio* [193, 194] have contributed extensively to elucidating the molecular basis of the disease, although mice are the predominantly used model. Each animal model has its own characteristics, and researchers must choose the one that is best suited to the research question. Among less complex animal models, zebrafish *Danio rerio* has become a popular model to study developmental processes and human diseases and now also plays a prominent role in cancer research [195]. The main advantages of this model are its elevated fecundity, the formation of optically transparent embryos that develop outside the mother, with most of the main organs conserved among the other vertebrates in terms of architectural and cellular features (e.g., brain and bone marrow niches are well formed) [193, 196–198]. In addition, the development of transgenic fish strains has proven an exceptional tool for the study of oncogenes [196, 197]. Zebrafish have been used to investigate the role of a vast number of genetic alterations occurring in human cancers, leading to the identification of potential driver genes. A widely used method for testing tumorigenicity is that of transplanting tumor cells in vivo in a recipient that belongs to the same species (allograft) or to another species (xenograft) [193]. This is usually obtained by injecting human cancer cells into immunocompromised mice, with limitations in the resolution of imaged cells and in the number of animals that can be used. The zebrafish model overcomes such limitations: the small size coupled with the optical transparency of the embryos allows the visualization of growing tumors with single-cell resolution [198, 199]. Zebrafish embryos can be grown in multiwell plates and this, coupled with their low cost, highlight their potential as models for high-throughput screening of new anticancer drugs to increase the power of statistical analysis [200, 201]. This leads to more robust and reliable data before moving to mammalian models.

**Fig. 5:**
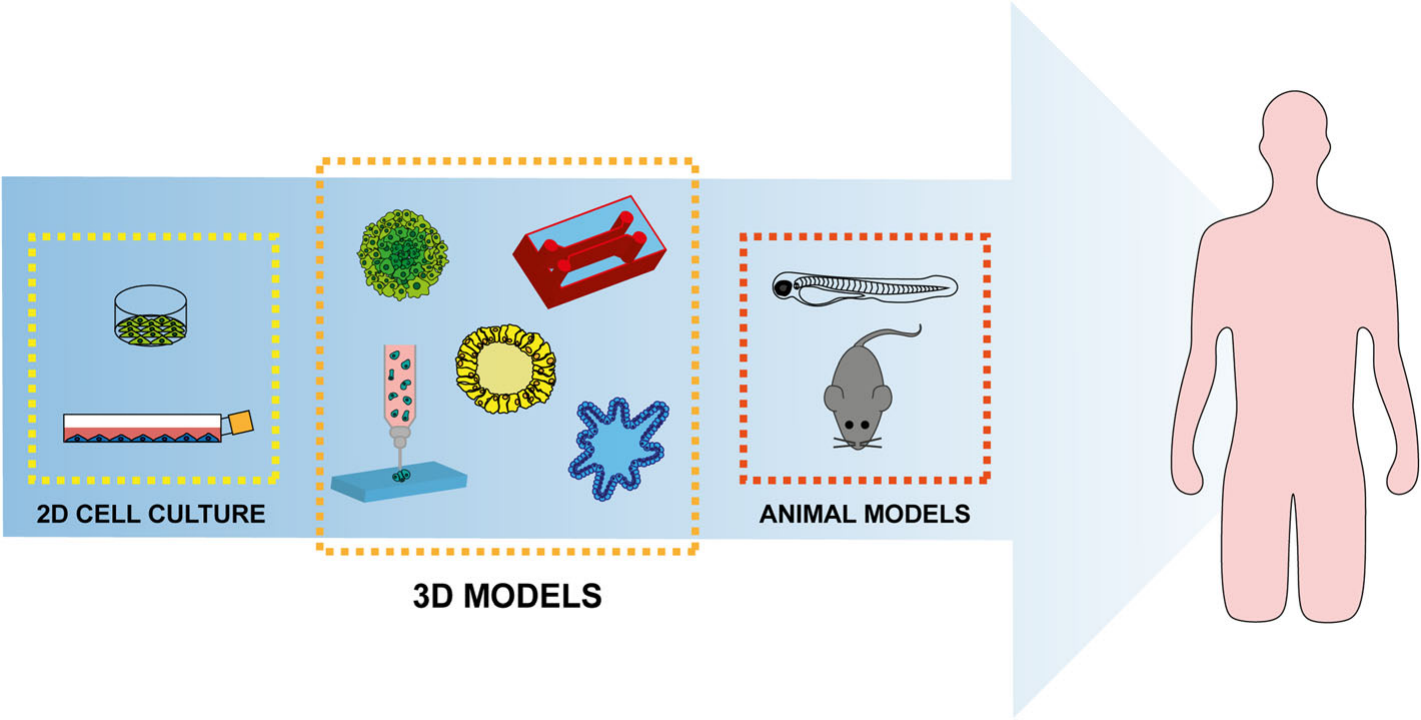
Current preclinical cancer research. 3D systems combined with new technologies such as organ-on-a-chip and 3D bioprinting could fill the gap between traditional 2D cell culture and animal models, producing more reliable data while also reducing costs, time to results, and political/ethical issues before their transition to clinical practice

Mice are the predominantly used animals among mammalian models, and PDXs in particular have emerged as an important and promising platform for research into new cancer treatments and biomarkers [16, 202]. In PDXs, surgically derived patient tumor samples are implanted into immunodeficient mice, maintaining the original proportion of tumor and stromal cells [16, 116]. These models closely resemble the clinical tumor architecture and the characteristics of each patient’s tumor, recapitulating the intertumor and intratumor heterogeneity of human cancers [16, 203, 204]. However, the use of only a small amount of the patient’s tumor tissue results in an incomplete representation of the original tumor [205]. Indeed, more than 50% of mutations found in primary cancer tissue may not be detected, while some new mutations may appear during the early passages in PDXs [206]. Furthermore, the contribution of stromal microenvironment in PDXs remains controversial. Human TME components such as immune cells, vasculature, and stromal cells only survive for a short time, eventually being replaced by murine stroma [207]. This limits the time in which TME crosstalk can be studied [116]. In addition to mouse-specific tumor evolution [7, 16, 18], another important limitations of PDX models is their limited engraftment efficiency based on tumor subtypes and grade [208]. Advanced tumor subclones may grow better than less advanced tumors in PDXs [209]. It is now known that orthotopic transplantation of tumor tissue provides more reliable PDXs than those obtained through heterotopic engraftment. In fact, there is evidence that the transplantation of primary tumor fragments in the corresponding anatomical position in mice leads to a local invasive behavior and the development of metastasis similar to that observed in humans [210, 211]. Metabolic differences were found in a comparison study between orthotopic and subcutaneous PDXs of pancreatic ductal adenocarcinoma, highlighting the importance of the location and the surrounding environment of the transplantation site [116, 212]. This approach remains time- and resource-consuming and should be reserved for validating robust in vitro data. Despite this, animal model experimentation still represents a crucial part of cancer studies aimed at understanding tumor biology and at finding innovative therapeutic strategies. The advent of 3D models could constitute an important step forward in cancer research, filling the gap between traditional cell cultures and animal models (Fig. 5) and helping to reduce the use of animals, especially in drug discovery and toxicity studies, before clinical trials in humans (Fig. 5). For now, animal models are still not fully replaceable and thus continue to be essential for cancer research.

## Conclusions

Cancer spheroids and organoids have proven to more closely resembling the pathophysiological features of clinical tumors than 2D cell cultures, approaching the level of in vivo models. In particular, organoid models retain the cellular and molecular phenotypes of original patient tumors, providing a powerful tool to investigate the onset of disease, progression, and biology and the development of more effective and personalized anticancer therapies. However, further efforts are needed to increase the degree of complexity of such models to develop more sophisticated systems that take into account all the cellular, physical, and biochemical components of the TME. Within this context, organ-on-a-chip and bioprinting technologies in combination with spheroid and organoid models could constitute an enormous step forward in the area of tumor modelling, marking an exciting new era in the landscape of preclinical cancer research (Fig. 5). Together, these systems unite the cellular complexity of organoids with the precise spatial organization provided by 3D bioprinting and the structural, physical, mechanical, and perfusion cues reconstructed on organ-on-a-chip systems. However, animal models remain indispensable, and advances have also been made in this area with the development of humanized murine models [213]. In this setting, the introduction of human hematopoietic stem cells (HSCs) into severely immunodeficient mice (i.e., NOD/SCID/IL2Rcnull (NSG) mice), coupled with the engraftment of human cancer cells, has enabled the dynamic growth of tumors to be investigated in vivo in the presence of a competent human immune system [214]. The combined use of 3D models could represent the next step forward in in vitro research into the development of effective anti-tumor treatments, also reducing animal testing and consequently costs, time to results, and political and ethical issues. Improvement in the robustness and reliability of research data would consequently follow, increasing their transferability from bench to bedside.

## Data Availability

Not applicable

## Abbreviations

3D: Three-dimensional
2D: Bidimensional
FDA: Food and Drug Administration
EVs: Extracellular vesicles
ECM: Extracellular matrix
TME: Tumor microenvironment
MTS: Multicellular tumor spheroids
MTDS: Multicellular tumor-derived spheroids
CSCs: Cancer stem cells
ALDH: Aldehyde dehydrogenase
ABC: ATP-binding cassette
CAFs: Cancer-associated fibroblasts
iPSCs: Induced pluripotent stem cells
PDXs: Patient-derived xenografts
CRC: Colorectal cancer
ROCK2: Rho-associated protein kinase 2
HSCs: Hematopoietic stem cells
NSG: NOD/SCID/IL2Rc null mice
